# CHD1L Inhibitor OTI-611 Synergizes with Chemotherapy to Enhance Antitumor Efficacy and Prolong Survival in Colorectal Cancer Mouse Models

**DOI:** 10.3390/ijms252313160

**Published:** 2024-12-07

**Authors:** Rita Sala, Hector Esquer, Timothy Kellett, Sophia Clune, Paul Awolade, Laura A. Pike, Qiong Zhou, Wells A. Messersmith, Daniel V. LaBarbera

**Affiliations:** 1 Department of Pharmaceutical Sciences, The Skaggs School of Pharmacy and Pharmaceutical Sciences, The University of Colorado Anschutz Medical Campus, Aurora, CO 80045, USA; 2 The CU Anschutz Center for Drug Discovery, Aurora, CO 80045, USA; 3 The University of Colorado Cancer Center, Aurora, CO 80045, USA; 4 Division of Medical Oncology, The School of Medicine, The University of Colorado Anschutz Medical Campus, Aurora, CO 80045, USA

**Keywords:** CHD1L, CHD1L inhibitor (CHD1Li), colorectal cancer, cancer, DNA damage response, PARthanatos, cancer drug combinations, chemotherapy, cancer pharmacology

## Abstract

Colorectal cancer (CRC) is one of the most prevalent and deadly forms of cancer. It is universally treated with a combination of the DNA damaging chemotherapy drugs irinotecan, 5-Fluorouracil (5-FU), and oxaliplatin. *CHD1L* is a novel oncogene that plays critical roles in chromatin remodeling and DNA damage repair, as well as the regulation of malignant gene expression. We show that an inhibitor of CHD1L, OTI-611, when combined with chemotherapy significantly increases DNA damage in CRC cell lines. OTI-611 also synergizes with SN-38, 5-FU, and oxaliplatin in killing CRC tumor organoids. We also demonstrate that, as in breast cancer, OTI-611 traps CHD1L, PARP1, and PARP2 onto chromatin. The entrapment of CHD1L causes the deprotection of PAR chains in the nucleus, ultimately resulting in cell death by CHD1Li-mediated PARthanatos, as measured by AIF translocation to the nucleus. Finally, the combination of low doses of OTI-611 with irinotecan significantly reduces tumor volume and extends survival in CRC xenograft mouse models compared to irinotecan alone. The combination of standard of care chemotherapy drugs with CHD1Li represents a promising advancement in future therapeutic strategies for CRC and other cancers driven by *CHD1L*.

## 1. Introduction

Colorectal cancer (CRC) is the third most diagnosed cancer each year and the second leading cause of cancer-related deaths worldwide, with metastasis being the primary cause of mortality [1,2]. Despite progress in prevention strategies that have lowered CRC incidence and mortality, nearly a quarter of patients are still diagnosed at an advanced or metastatic stage, where the 5-year survival rate is just 13% [2,3]. Chemotherapy, which typically includes a combination of irinotecan, 5-fluorouracil (5-FU), and oxaliplatin, is a standard of care (SOC) treatment for CRC and is expected to remain so for the foreseeable future [4,5,6]. However, only about 50% of patients initially respond to chemotherapy and most will eventually develop resistance [7].

Several well-known mechanisms of resistance to CRC chemotherapy have been identified [8]. These include increased drug efflux through the overexpression of ATP-binding cassette (ABC) transporters such as P-glycoprotein, which reduces intracellular drug accumulation [9]. Additionally, alterations in drug targets, such as mutations in thymidylate synthase and topoisomerase I, contribute to resistance against 5-FU and irinotecan, respectively [10,11]. Enhanced DNA damage repair also confers resistance, especially to irinotecan and oxaliplatin [12,13]. Furthermore, cancer cells can evade programmed cell death mechanisms such as apoptosis and the immune response [14].

**Figure 1:**
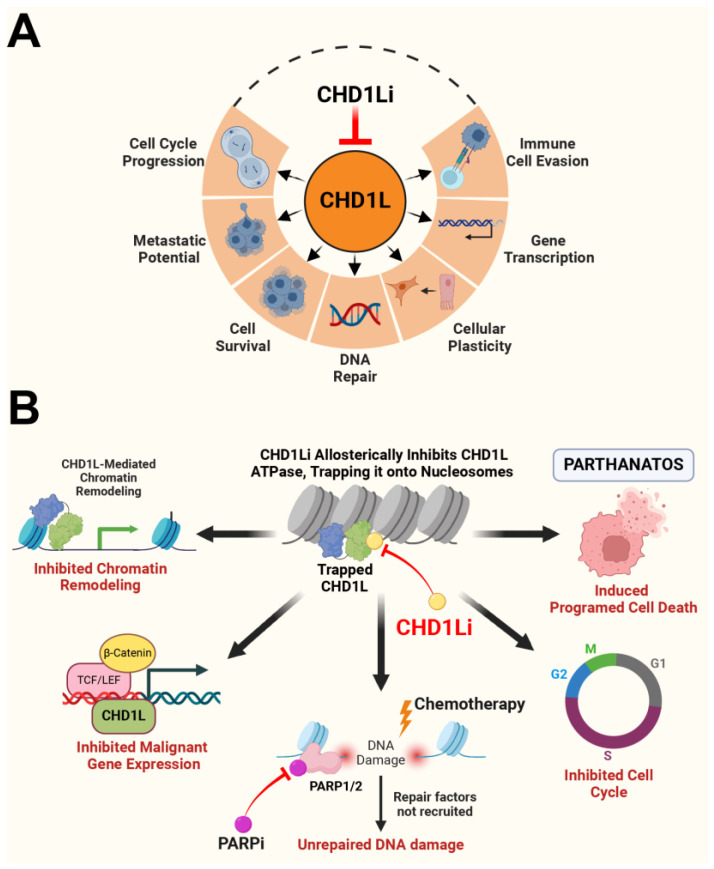
Oncogenic functions of CHD1L and the mechanism of action of CHD1Li. (**A**) A cartoon representation of the oncogenic functions of CHD1L. (**B**) The mechanism of action (MOA) of CHD1Li. CHD1Li allosterically inhibits CHD1L, trapping it on chromatin and blocking chromatin remodeling. This entrapment suppresses malignant gene expression, disrupts cancer cell survival pathways, including DDR, and induces PARthanatos, a unique form of programmed cell death.

Chromodomain Helicase DNA Binding Protein 1-Like (CHD1L), also known as ALC1, is a chromatin remodeling enzyme that has emerged as an oncogenic protein with diverse biological functions (Figure 1A) [15]. Elevated *CHD1L* expression in patients is linked to poor prognosis, accelerated disease progression, metastasis, and decreased survival rates across multiple cancers, including CRC [15,16,17]. Notably, *CHD1L* is a key regulator of tumor survival pathways, including the cell cycle, DNA damage response and repair (DDR), and the inhibition of programmed cell death. Recently, we reported that CHD1L is a master regulator of PARthanatos [18], a unique form of programmed cell death mediated by poly-ADP ribose (PAR) and apoptosis inducing factor (AIF) [19,20]. Collectively, CHD1L’s oncogenic functions pose a formidable challenge to SOC chemotherapy and targeted drug therapies for the treatment of CRC and other cancers. Therefore, small molecule inhibitors of CHD1L (CHD1Li) may be an effective strategy to treat cancer, particularly when used as a combination therapy with SOC chemotherapy and other targeted drug therapies.

We previously discovered and optimized the first CHD1Li, leading to the development of OTI-611 as a potential lead drug for CRC [21]. Recently, we characterized the antitumor mechanism of action of CHD1Li using OTI-611 (Figure 1B). OTI-611 binds allosterically to CHD1L, inhibiting its ATPase activity. This traps CHD1L on chromatin, preventing chromatin relaxation and blocking access to DDR proteins at DNA damage sites. Once CHD1L is trapped, it can no longer bind to PAR chains, allowing auto-PARylated PARP1/2 to undergo PAR chain hydrolysis, which further traps PARP1/2 on chromatin. Unlike PARP inhibitors (PARPi), which trap PARP1/2 at DNA damage sites, causing replication fork collapse and DNA damage, CHD1Li-mediated entrapment of CHD1L and PARP1/2 halts DDR and cell cycle progression without inducing DNA damage. This leaves PAR chains exposed for PARG-mediated hydrolysis, ultimately leading to PARthanatos [18].

The unique mechanism of action of CHD1Li offers a promising complement to the SOC chemotherapy for CRC. While SOC chemotherapy induces DNA damage to kill cancer cells, CHD1Li works by preventing DDR and cell cycle progression without introducing additional DNA damage. By trapping CHD1L and disrupting chromatin remodeling, CHD1Li blocks the repair mechanisms that cancer cells rely on to survive chemotherapy-induced damage. This combination strategy has the potential to enhance the efficacy of SOC treatments by inhibiting tumor progression, sensitizing cancer cells to chemotherapy and other targeted therapy, reducing their ability to repair, and ultimately driving them toward programmed cell death, including PARthanatos. This synergistic approach could improve outcomes for patients with CRC, especially in cases where resistance to traditional therapies develops. In this study, we demonstrate the synergy between the pharmacological inhibition of CHD1L by OTI-611 and CRC SOC chemotherapy, both in vitro and using xenograft models. Moreover, we also unravel the mechanism behind the synergistic effect leading to non-apoptotic cell death through PARthanatos.

## 2. Results

### 2.1. CHD1L Inhibition Potentiates DNA Damage in CRC Cell Lines

**Figure 2:**
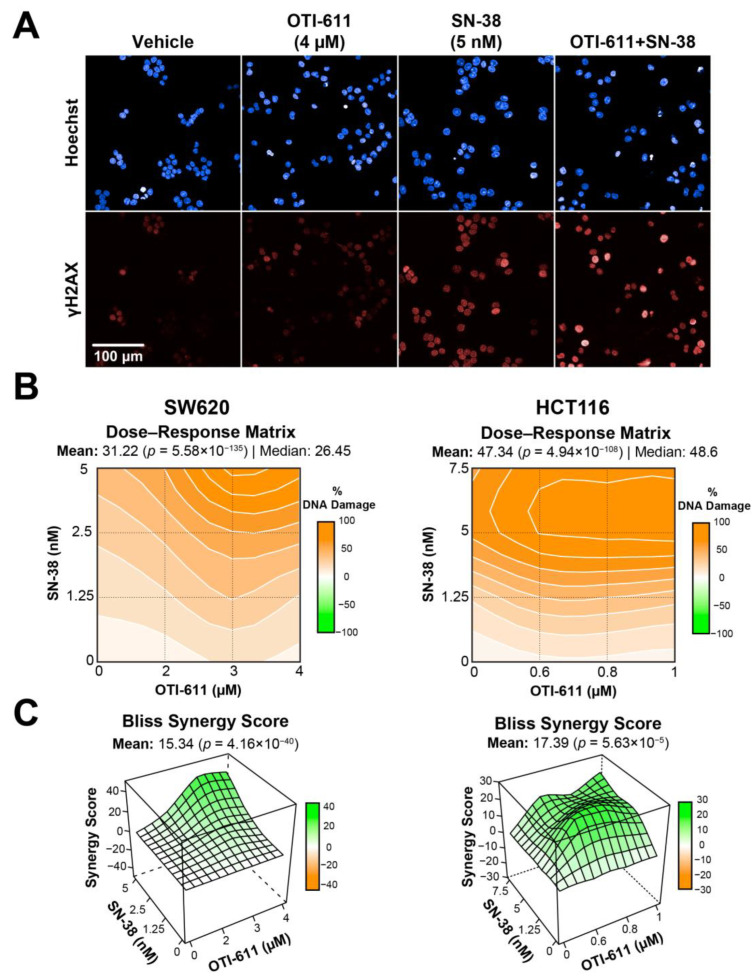
OTI-611 combined with SN38 potentiates DNA damage in CRC. (**A**) Representative images of γ-H2AX immunofluorescence in SW620 cells treated for 24 h with OTI-611, SN-38, and its combination. Scale bar = 100 µm. (**B**) Dose–response matrices representing the percentage of DNA damage measured by γ-H2AX immunofluorescence for SW620 and HCT116 cells treated with OTI-611, SN-38, and its combination for 24 h. (**C**) Bliss Synergy 3D plots showing synergy scores for each dose combination of OTI-611 and SN-38 in SW620 and HCT116 cells. Bliss Synergy scores were generated using the SynergyFinder R package. Synergistic drug interaction is considered when values above 10. Data presented as mean of two independent experiments ± S.E.M with each independent experiment performed in duplicate technical replicates.

Irinotecan is a DNA damaging drug that is commonly used as a chemotherapeutic agent in the treatment of CRC. The active metabolite of irinotecan, SN-38, is a potent topoisomerase 1 (TOP1) poison, which stabilizes the TOP1-DNA complex, leading to DNA replication fork collision and double-strand DNA breaks [22]. As CHD1L is known to be implicated in the DDR by remodeling the chromatin, we hypothesized that the inhibition of CHD1L should potentiate DNA damage caused by DNA damaging drugs in CRC. Our hypothesis is further supported by similar findings observed in breast cancer, where OTI-611 synergized with Olaparib and Doxorubicin treatments [18]. To test our hypothesis, we used immunofluorescence to visualize phosphorylated H2AX (γ-H2AX) in SW620 and HCT116 cells treated with SN-38, OTI-611, or both agents in combination for 4 or 24 h. We observed significantly higher γ-H2AX intensity or an increased number of foci in the cells treated with the combination than in cells treated with either single agent alone (Figure 2A,B and Supplemental Figure S1). Bliss Synergy analysis of the data provided a synergy score of 15.34 for SW620 cells and 17.39 for HCT116 cells, with *p*-values under 0.05, indicating statistically significant synergy (Figure 2C).

**Figure 3:**
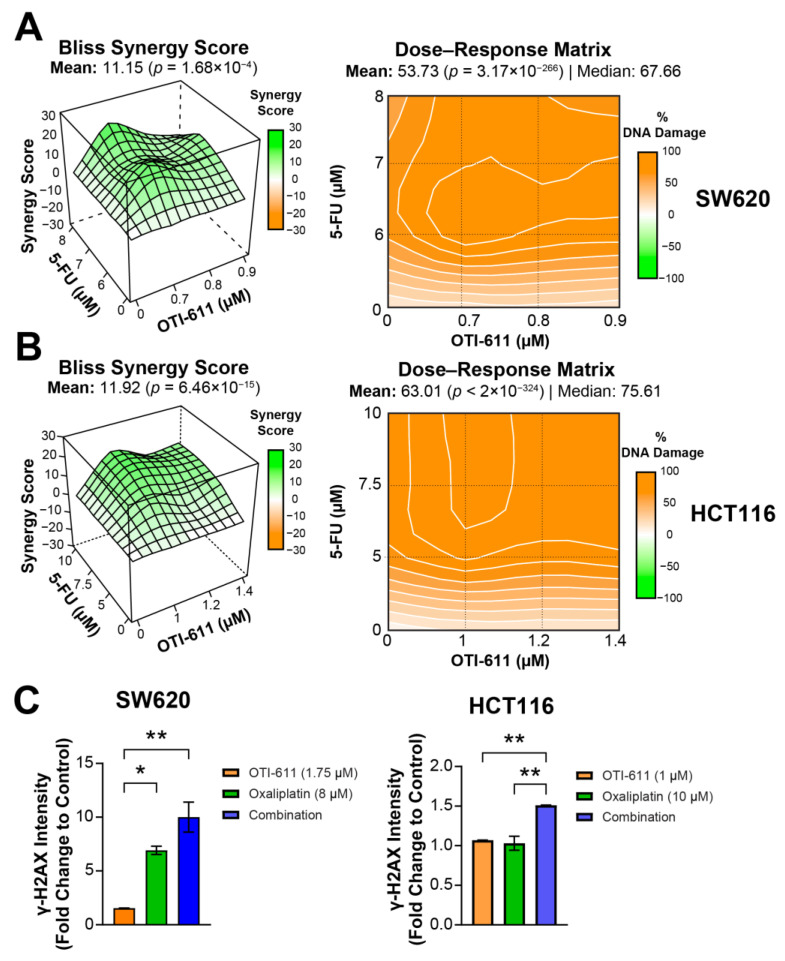
OTI-611 combined with 5-FU or oxaliplatin potentiates DNA damage in CRC. (**A**) Bliss Synergy 3D plot and dose–response matrix showing OTI-611′s synergistic effect on γH2AX foci in combination with 5-FU in SW620 cells. (**B**) Bliss Synergy 3D plot and dose–response matrix showing OTI-611′s synergistic effect on γH2AX foci in combination with 5-FU in HCT116 cells. (**C**) Bar graphs representing change in γH2AX after treatment with OTI-611, oxaliplatin, or combinations in SW620 (left) and HCT116 (right) cells. Data presented as mean of two independent experiments ± S.E.M with each independent experiment performed in duplicate technical replicates. * *p* < 0.05, ** *p* < 0.01.

We also evaluated the possible synergistic effect of CHD1L inhibition with other drugs used in the treatment of CRC such as 5-Fluorouracil (5-FU) or oxaliplatin. 5-FU causes DNA damage due to the misincorporation of FdUTP into DNA, and this damage is potentiated when combined with OTI-611 in both SW620 and HCT116 cell lines. For the combination of OTI-611 and 5-FU in SW620 and HCT116, the Bliss Synergy scores were 11.15 and 11.92, respectively (Figure 3A,B and Supplemental Figure S2). On the other hand, OTI-611 also potentiates the DNA damage caused by oxaliplatin by 3-fold in SW620 and 1.5-fold in HCT116 cell lines (Figure 3C and Supplemental Figure S3). These results suggest that CHD1Li can synergize with a variety of DNA damaging agents, underscoring the importance of CHD1L in DDR mechanisms.

### 2.2. CHD1L Inhibition Synergizes with SOC Chemotherapy in CRC Tumor Organoids

**Figure 4:**
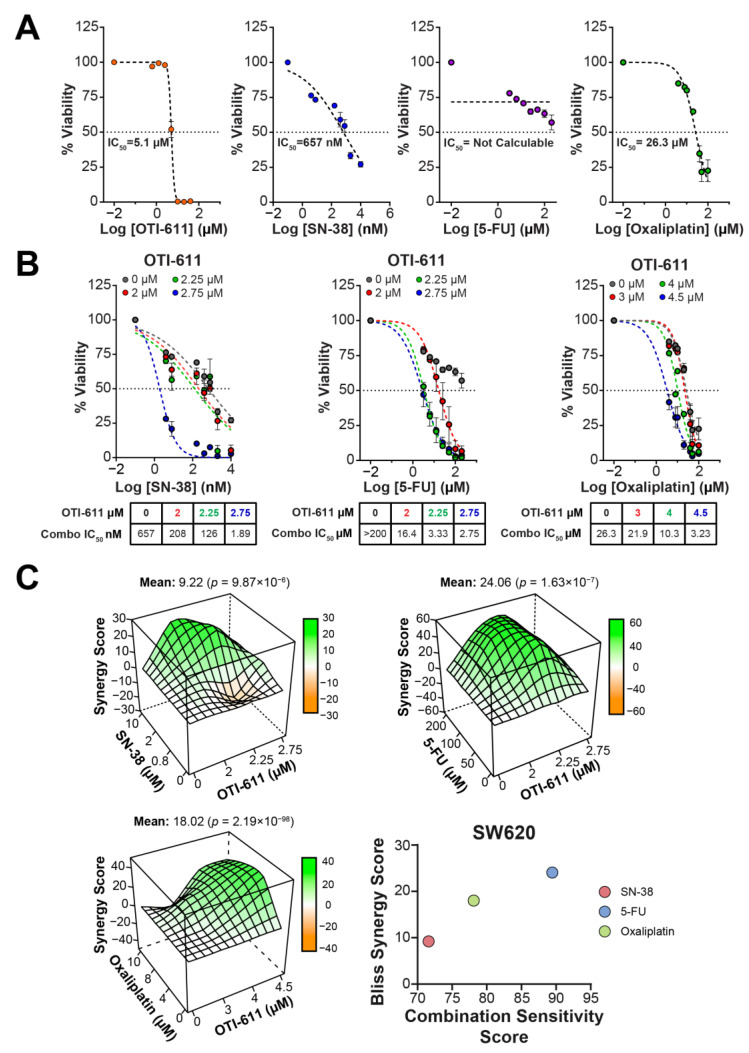
OTI-611 synergizes with SOC in SW620 tumor organoids. (**A**) Single agent dose–response curves with calculated IC_50_ values of OTI-611, 5-FU, oxaliplatin, and SN-38. (**B**) Dose–response curves of drug combinations with OTI-611. (**C**) Three-dimensional contour plot of Bliss Synergy score values and scatter plot of Bliss Synergy score vs. Combination Sensitivity score as calculated using SynergyFinder R-package of drug combinations. Data shown as SEM of triplicate experiments in triplicate of conditions.

**Figure 5:**
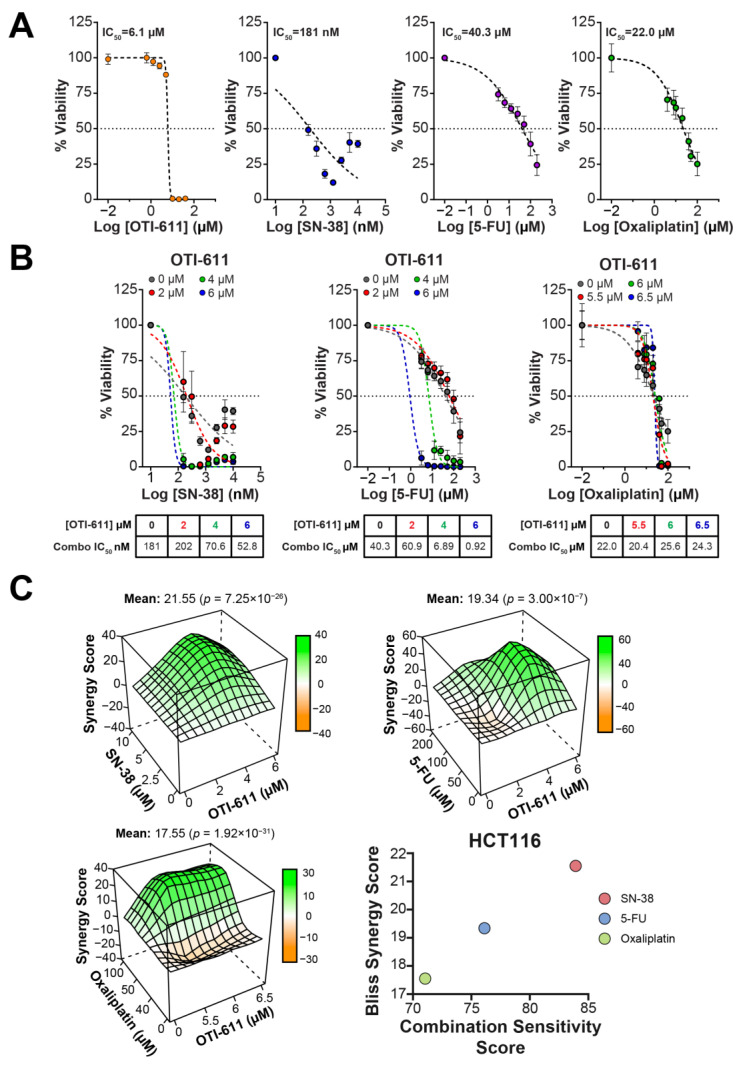
OTI-611 synergizes with SOC in HCT116 tumor organoids. (**A**) Dose–response curves with calculated IC_50_ values of OTI-611, 5-FU, oxaliplatin, and SN-38. (**B**) Dose–response curves of drug combinations with OTI-611. (**C**) Three-dimensional contour plot of Bliss Synergy score values as calculated using SynergyFinder R-package of drug combinations and scatter plot displaying Bliss Synergy score values vs. Combination Sensitivity score values of the various drug combinations. Data shown as SEM of triplicate experiments in triplicate of conditions.

**Figure 6:**
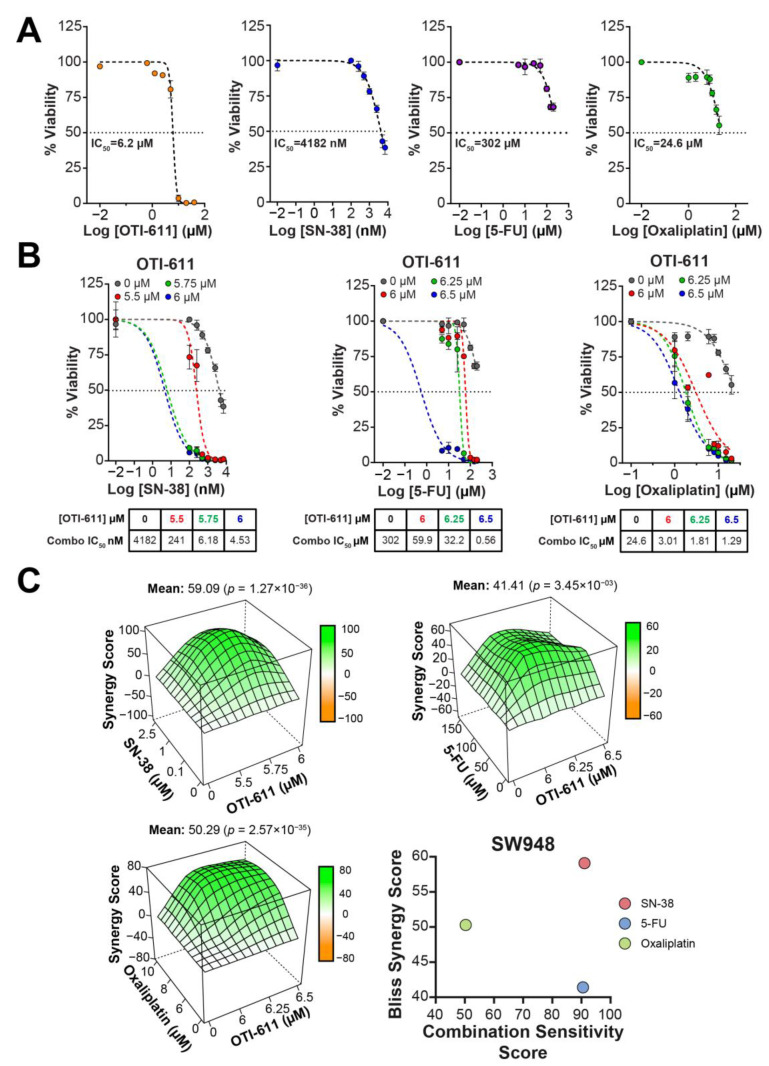
OTI-611 synergizes with SOC in SW948 tumor organoids. (**A**) Single agent dose–response curves with calculated IC_50_ values of OTI-611, 5-FU, oxaliplatin, and SN-38. (**B**) Dose–response curves of drug combinations with OTI-611. (**C**) Three-dimensional contour plot of Bliss Synergy score values as calculated using SynergyFinder R-package of drug combinations and scatter plot displaying Bliss Synergy score values vs. Combination Sensitivity score values of the various drug combinations. Data shown as SEM of triplicate experiments in triplicate of conditions.

Following our results indicating that OTI-611 synergizes with SOC chemotherapy to cause DNA damage, we tested if this effect also translates to increased cytotoxicity in CRC tumor organoids. OTI-611 is highly cytotoxic to tumor organoids, potently inhibiting viability with half maximal inhibitory concentration (IC_50_) values of 5.1 µM in SW620 organoids, 6.1 µM in HCT116 organoids, and 6.2 µM in SW948 organoids (Figure 4A, Figure 5A and Figure 6A). When combining sub-lethal doses of OTI-611 with SOC chemotherapy (SN-38, 5-FU, and oxaliplatin), we observed a synergistic cytotoxic effect with each of the drugs in all three models (Figure 4, Figure 5 and Figure 6). In SW620 tumor organoids, the combination improved the IC_50_ potency of SN-38 by almost 350-fold from 657 nM to only 1.9 nM thus rendering cells more sensitive to SN-38.

The synergistic effect was also pronounced in 5-FU-treated organoids, which exhibited little sensitivity to 5-FU as a single agent. OTI-611 improved the IC_50_ potency of 5-FU by more than 70-fold with a Bliss Synergy score of 24 (Figure 4B,C). Lastly, the inhibition of CHD1L also synergized with oxaliplatin by decreasing its IC_50_ 8-fold from 26.3 µM to 3.2 µM, with a Bliss Synergy score of 18. HCT116 tumor organoids are highly sensitive to SN-38 with a single agent IC_50_ of 181 nM, and the combination with OTI-611 further increases the sensitivity by 3.4-fold (53 nM) (Figure 5). HCT116 organoids treated with 5-FU decreased the IC_50_ value of the single agent from 40.3 µM to 0.9 µM in combination, a 44.8-fold increase in cytotoxic potency. Meanwhile, in oxaliplatin treated HCT116 organoids, the IC_50_ values were not significantly reduced by CHDLi, but a Bliss Synergy score of 17.55 and a calculated Combination Sensitivity Score (CSS) of 71 indicate synergy (Figure 5).

CSS values signify the combination efficacy, with a maximum possible score of 100 (Figure 4C, Figure 5C and Figure 6C). The scores are calculated by comparing the area under the curve (AUC) of the calculated cytotoxicity curves, where one drug is kept constant at its IC_50_ while varying the concentration of the other drug. CSS values add valuable information in the context of combination studies by providing a metric that easily reflects the sensitivity to the tested drug combination [23]. For example, two drugs can have high synergy values, but if their CSS value is low, it indicates that the cell death response is moderate to low. Interestingly, SW948 tumor organoids are more resistant to SN-38 treatment with an IC_50_ value of 4.182 µM, which is reduced to 4.53 nM, a 923-fold increase in potency compared to SN-38 alone (Figure 6B). SW948 cells are also resistant to 5-FU (IC_50_~302 µM), which, when combined with OTI-611, the IC_50_ is reduced to 0.56 µM (a 539-fold estimated increase in potency). Finally, oxaliplatin-treated SW948 tumor organoids displayed an IC_50_ value of 24.6 µM and a drug combination potency increase to an IC_50_ value of 1.29 µM, a 19.1-fold change (Figure 6B). Taken together, the drug potency improvement, synergy, and CSS values reveal the versatility of CHD1Li in CRC lines derived from different patients and with different mutational backgrounds.

### 2.3. Pharmacological Inhibition of CHD1L Traps CHD1L and PARP1/2 onto Chromatin

**Figure 7:**
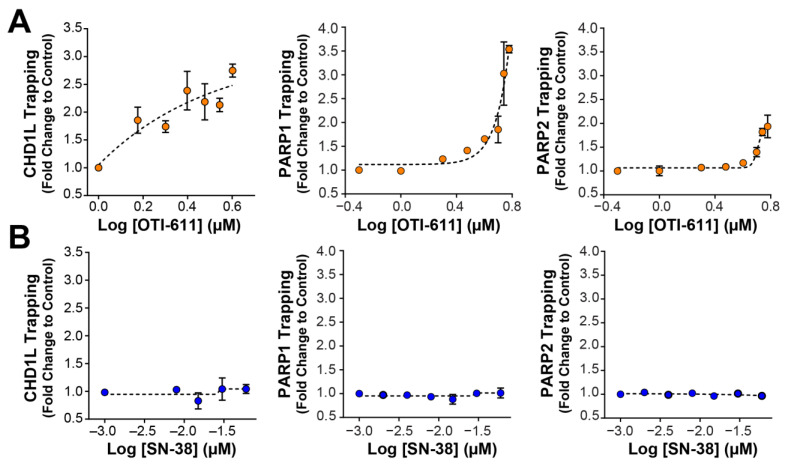
OTI-611 traps CHD1L, PARP1, and PARP2 onto chromatin. (**A**) Trapping profiles of CHD1L, PARP1, and PARP2 measured after dose–response treatment with OTI-611. (**B**) Trapping profiles of CHD1L, PARP1, and PARP2 measured after dose–response treatment with SN-38. For all conditions, SW620 cells treated with drug of interest in combination with 0.001% MMS for 4 h. All data normalized to MMS only treated cells and expressed as mean of two independent experiments ± S.E.M in duplicate technical replicates.

CHD1L plays an important role in chromatin remodeling and relaxation, rendering DNA more accessible to other repair factors. Upon DNA damage, CHD1L’s macro domain binds to the PAR chains on the histones produced by PARP and HPF1, and the catalytically active conformation is stabilized by the binding of the regulatory linker region to the H2A-H2B acidic patch of the nucleosome. Then, the ATPase domain uses ATP to slide the nucleosomes and expose the DNA lesions [24,25]. We have previously reported in breast cancer cell lines that the pharmacological inhibition of the CHD1L ATPase activity by OTI-611 traps CHD1L onto chromatin [18]. As is consistent with our previous results, OTI-611 trapped CHD1L onto chromatin in SW620 cells in a dose-dependent manner (Figure 7A), while SN-38 did not trap CHD1L (Figure 7B). CHD1L entrapment causes the deprotection of PAR chains in the nucleus and their PARG-mediated hydrolysis [18]. Thus, CHD1L inhibition by OTI-611 indirectly causes PARP1/2 trapping due to the hydrolysis of PAR chains, which are necessary for their release from DNA (Figure 7A). Again, SN-38 did not show any PARP1/2 trapping when treating the cells at different concentrations (Figure 7B). These results suggest that OTI-611-induced CHD1L and PARP1/2 trapping are not specific to breast cancer and can also be observed in CRC tumors.

### 2.4. Inhibition of CHD1L Induces PARthanatos Through AIF Translocation to Nucleus

**Figure 8:**
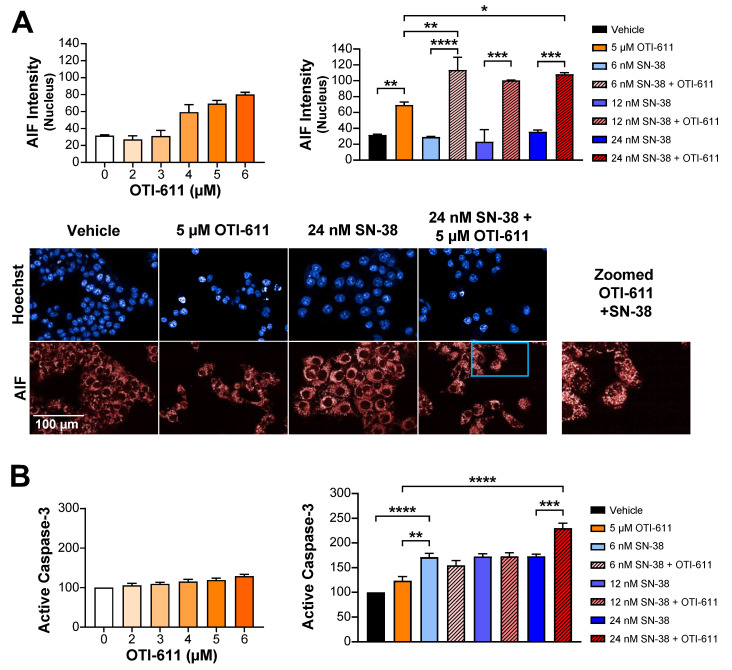
Induction of PARthanatos cell death by OTI-611. (**A**) Intensity of nuclear AIF in HCT116 cells treated for 18 h with OTI-611, SN-38, and its combination. Representative images of AIF immunofluorescence also shown. Scale bar = 100 µm. AIF mean intensity normalized to vehicle and expressed as mean of two independent experiments ± S.E.M. (**B**) Intensity of active caspase-3 measured in HCT116 cells treated for 18 h with OTI-611, SN-38, and its combination. Active caspase-3 mean intensity normalized to vehicle and expressed as mean of three independent experiments ± S.E.M in duplicate technical replicates. * *p* < 0.05, ** *p* < 0.01, *** *p* < 0.001, **** *p* < 0.0001.

We then examined the mechanism of cell death induced by a combination treatment of SN-38 and OTI-611. We have previously described that OTI-611 increases PAR translocation to the cytoplasm, triggering the release of AIF from the mitochondria and its translocation to the nucleus: a key step in the cell death mechanism of PARthanatos [18]. Here, we sought to confirm the involvement of PARthanatos in the cytotoxic synergistic effect observed in CRC tumor organoids when treated with a combination of SN-38 and OTI-611. We observed a dose-dependent translocation of AIF into the nucleus in HCT116 cells treated for 18 h with up to 6 µM of OTI-611 (Figure 8A). SN-38 alone did not induce AIF translocation to the nucleus but when combined with 5 µM of OTI-611 we observed a significantly higher translocation as compared to either drug alone (Figure 8A). Thus, this suggests a synergistic interaction between SN-38 and OTI-611 through the induction of PARthanatos. Finally, to further validate that the main mechanism of cell death triggered by OTI-611 is PARthanatos, we assessed the activation of caspase-3 as a marker of apoptosis (Figure 8B and Supplemental Figure S4). OTI-611 caused no significant change in active caspase-3 levels compared to the control, while all SN-38 doses substantially increased its activation, which led to an apoptotic cell death. Interestingly, only the combination of OTI-611 and the highest dose of SN-38 (24 nM) significantly increased the levels of active caspase-3. In contrast, all tested doses of SN-38 when combined with 5 µM of OTI-611 induced AIF translocation to the nucleus (Figure 8A). Therefore, OTI-611 shifts the SN-38-induced cell death mechanism from apoptosis to PARthanatos, which suggests that this type of programmed cell death is a key mechanism induced by the pharmacological inhibition of CHD1L with OTI-611.

### 2.5. OTI-611 Synergizes with Irinotecan in a CRC Xenograft Model

**Figure 9:**
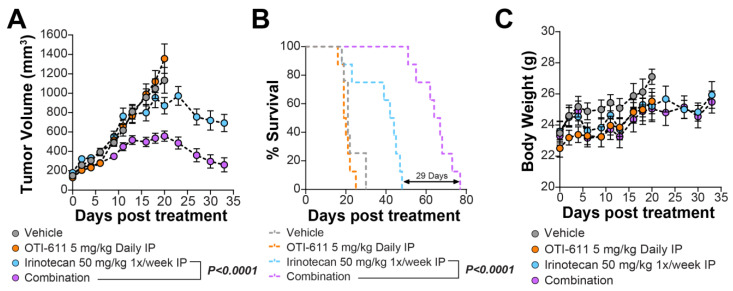
OTI-611 synergizes with irinotecan in CRC xenograft models. (**A**) Average tumor volume over time in each group. Combination-treated mice had significantly lower average tumor volume by the end of the study than single agent or vehicle groups (*p* < 0.0001). (**B**) Percentage survival over time in each group. On average, the group receiving OTI-611 with irinotecan combination lived 29 days longer than mice receiving irinotecan alone, second longest-living group (*p* < 0.0001). (**C**) Average body weight over time in each group. No significant difference between groups as calculated by 2-way ANOVA.

To assess the efficacy of OTI-611 combined with irinotecan in vivo, SW620 isolated mesenchymal cells were injected into the flank of nude athymic mice and allowed to grow for 2 weeks (165 mm^3^ average volume). The irinotecan dose was selected based on literature reports, suggesting that 100 mg/kg, administered intraperitoneally once weekly for four weeks, is an effective antitumor regimen [26]. Based on the strong synergy observed in cell-based models, we reduced the dose by half to 50 mg/kg weekly in our experiments to ensure a significant combination effect with OTI-611. Likewise, the OTI-611 dose was determined from previous studies showing that 50 mg/kg administered intraperitoneally is readily absorbed into the plasma. For this study, the dose was reduced 10-fold to 5 mg/kg to accommodate a daily dosing regimen aimed at capturing the synergistic effects of the combination in vivo. The mice were then randomized and treated with either vehicle, 5 mg/kg of OTI-611, 50 mg/kg of irinotecan, or the combination of OTI-611 and irinotecan. OTI-611 was administered intraperitoneally (IP) daily, and irinotecan was administered IP once per week (Figure 9). The results show that the combination group had significantly reduced tumor volume on day 33 compared to either OTI-611 (*p* = 0.028) or irinotecan (*p* = 0.0009) alone (Figure 9A). Tumor growth was not inhibited by the OTI-611 single agent, while 30% was inhibited by irinotecan and 70% by the combination. Additionally, the combination group experienced significantly longer survival (77 days, *p* < 0.0001) than either OTI-611 (25 days) or irinotecan (48 days) alone (Figure 9B). Over the course of treatment, the body weights of mice did not differ significantly between the groups, indicating tolerability (Figure 9C). Our laboratory has previously demonstrated that single-agent OTI-611 significantly reduces tumor volume in a CRC tumor xenograft model [21]. Building on these findings, the objective of this study was to evaluate the in vivo synergy between OTI-611 and irinotecan. Specifically, we aimed to determine whether the combination could achieve greater tumor suppression and improved mouse survival compared to either agent alone. The results indicate that combining CHD1Li with chemotherapy produces a synergistic antitumor effect, supporting the potential of this therapeutic strategy for enhanced cancer treatment.

## 3. Discussion

Our previous work validated CHD1L as a significant oncogenic factor in CRC and its pharmacological inhibition as a targeted therapeutic strategy with the potential to overcome the limitations of conventional chemotherapy [17]. In this study, we provide compelling evidence that CHD1Li OTI-611 synergizes with SOC chemotherapeutics, including SN-38, 5-FU, and oxaliplatin, leading to unprecedented enhancements in their cytotoxic potency. Notably, using SW620 tumor organoids, we observed a 350-fold increase in cytotoxic potency for SN-38 and a 70-fold increase for 5-FU when combined with OTI-611. Furthermore, in the chemoresistant SW948 organoids, the IC_50_ values for SN-38 and 5-FU were reduced from ~4 µM to ~4 nM and from ~300 µM to ~500 nM, respectively, indicating an impressive 1000-fold and 500-fold increase in sensitivity compared to chemotherapy alone. The Bliss Synergy scores, consistently exceeding the threshold of 10 across combinations, affirm the substantial synergistic interactions between OTI-611 and these chemotherapeutic agents. This analysis not only validates the cooperative effects but also demonstrates that the combination therapies can effectively target resistance mechanisms commonly observed in CRC, including those related to the DDR and programmed cell death pathways [27]. Our previous and current findings elucidate the role of *CHD1L* in driving chemotherapy resistance and how OTI-611 overcomes these mechanisms to enhance therapeutic efficacy (Figure 1) [17,18,21].

Previous studies have shown that CHD1L depletion increases sensitivity to the DNA alkylating agent MMS (methyl methanesulfonate) and hydrogen peroxide [28]. However, the mechanism underlying the observed synergistic effect of pharmacological inhibition differs from gene depletion. Our data suggest that the OTI-611-mediated trapping of CHD1L on chromatin also results in PARP1/2 trapping, the inhibition of H2AX phosphorylation, and the disruption of the DDR signaling pathway. Furthermore, unlike PARPi, which traps PARP1/2 at exposed DNA damage sites and potentiates DNA damage [29], OTI-611-mediated entrapment of CHD1L prevents the chromatin remodeling required to expose these sites for repair [18]. As a result, OTI-611 inhibits DDR without inducing DNA damage itself while enhancing the DNA-damaging effects of chemotherapy. This mechanistic difference may explain the stronger sensitization observed with pharmacological CHD1L inhibition compared to gene knockout.

Our seminal report established CHD1L as a master regulator and inhibitor of PARthanatos [18], a distinct form of programmed cell death [19,20]. Prior to this finding, PARthanatos was understood to be triggered by genomic instability and excessive oxidative stress, which could be mitigated by the depletion of PARP1/2 or through the use of PARPi [19,30,31]. However, our previous work and the current study demonstrate that the entrapment of CHD1L onto chromatin by CHD1Li OTI-611 prevents CHD1L from protecting PAR chains. This deprotection caused by CHD1Li leads to the fragmentation of PAR chains by enzymes such as PARG, ultimately inducing PARthanatos, with the observed translocation of AIF to the nucleus serving as a hallmark of this process. The induction of PARthanatos by CHD1Li is not abrogated by PARPi, and PARPi do not induce PARthanatos themselves [18]. Furthermore, our findings indicate that OTI-611 not only enhances the effects of chemotherapy but also shifts the cell death modality from apoptosis to PARthanatos thereby improving the overall therapeutic impact. Taken together, our results highlight the unique mechanism of action of CHD1Li OTI-611.

We have validated our in vitro findings using CRC xenograft models, which yielded compelling in vivo evidence. The combination of OTI-611 and irinotecan significantly reduced tumor volume and prolonged survival in mice compared to treatment with irinotecan alone. These results reinforce the clinical relevance of our preclinical data and suggest that integrating OTI-611 with SOC treatments represents an effective strategy for overcoming chemoresistance in CRC patients. By improving the effectiveness of front-line clinical therapies, combinations involving CHD1Li could lead to better treatment outcomes and increased survival rates for patients with CRC and other cancer types.

## 4. Materials and Methods

### 4.1. Reagents and Antibodies

SN-38 (Cat. No. S4908, SelleckChem, Houston, TX, USA). 5-Fluorouracil (Cat. No. S1209, SelleckChem). Oxaliplatin (Cat. No. S1224, SelleckChem). MMS (Cat. No. AC156890050, ThermoFisher, Waltham, MA, USA). Phospho-Histone H2AX antibody (Cat. No. 4642S, Cell Signaling Technology, Danvers, MA, USA). CHD1L antibody (Cat. No. PA5-55940, ThermoFisher). PARP1 antibody (Cat. No. WH0000142M1, Sigma-Aldrich, Burlington, MA, USA). PARP2 antibody (Cat. No. 39004, Active Motif, Carlsbad, CA, USA). AIF antibody (Cat. No. 9718S, Cell Signaling Technology). Cleaved Caspase-3 antibody (Cat. No. 9661, Cell Signaling Technology). Goat anti-rabbit IgG Alexa Fluor Plus 647 (Cat. No. A32733, ThermoFisher). Goat anti-mouse IgG Alexa Fluor Plus 488 (Cat. No. A32723, ThermoFisher). Hoechst 33342 (Cat. No. H3570, ThermoFisher).

### 4.2. Cell Culture

Cell lines were STR-profiled and mycoplasma-tested prior to all experiments. HCT116 and SW620 cell lines were obtained and cultured as recommended by the American Type Culture Collection (ATCC). In addition, cells were grown in RPMI-1640 media (Cat. No. 11875119, ThermoFisher) supplemented with 5% fetal bovine serum (FBS) and maintained in 10 cm culture dishes in a humidified incubator at 37 °C and 5% CO_2_.

### 4.3. Cell Line Tumor Organoid Culture and Cytotoxicity 

Cells were plated at 2000 cells/well into 96-well Clear Round Bottom Ultra-Low Attachment plates (Cat. No. 7007, Corning, Corning, NY, USA) in complete growth medium. Cell assay plates were centrifuged at 1000 rpm for 15 min to promote cellular aggregation, then 25 µL of cold Growth-Factor Reduced (GFR) Matrigel (Corning) diluted in complete media were added per well for a final concentration of 2%. The tumor organoids were then allowed to grow for 72 h, and then treated with OTI-611, SN-38, 5-FU, and oxaliplatin for an additional 72 h. Cell viability was assessed by carefully transferring organoids to 96-well Cellstar white solid bottom plates (Cat. No. 655083, Greiner, Monroe, NC, USA) and incubating with an equal volume of CellTiter-Glo 3D (Cat. No. G9683, Promega, Madison, WI, USA) for 45 min on an orbital shaker at 450 rpm. CellTiter-Glo 3D’s luminescence signal was measured using the EnVision microplate reader (Revvity, Waltham, MA, USA) and values normalized to the control (vehicle) as 100% viability using GraphPad Prism 10 (GraphPad Software Inc., San Diego, CA, USA).

### 4.4. Confocal Imaging

Cells were imaged using the Opera Phenix Plus High-Content Screening (HCS) System (Revvity). A 40× water objective (NA 1.1) was used in confocal mode to image cells plated into 384-well or 96-well PhenoPlates (Revvity). The following fluorescent channels (Ex/Em) were used for immunofluorescence experiments: Alexa Fluor 647 ((640/650-760) Life Technologies, Carlsbad, CA, USA) and Hoechst 33342 ((405/435-480) ThermoFisher). Brightfield images were also acquired. A total of 25 fields of view per well were imaged and analyzed using Harmony 5.1 software (Revvity).

### 4.5. DNA Damage

SW620 and HCT116 cells were seeded into 96-well PhenoPlates (Revvity) and allowed to adhere overnight. The cells were then treated with OTI-611 or OTI-611 in combination with SN-38, 5-FU, or oxaliplatin for 4 or 24 h depending on the drug. The medium was then aspirated, and the cells were fixed with 4% paraformaldehyde for 15 min at room temperature (RT). After washing twice with PBS, the cells were incubated in blocking buffer (5% BSA, 0.3% Triton X-100 in PBS) for 30 min. Afterwards, the cells were washed again with PBS, incubated with anti-γH2AX primary antibody ((Cell Signaling, Danvers, MA, USA, cat #9718) 1:500, overnight (O/N), 4 °C)), and washed and incubated with a secondary antibody for 1 h at RT (anti-rabbit AlexaFluor 647 (Life Technologies), 1:500). Finally, the cells were washed, stained with Hoechst 33342 (1:1000), and imaged using a 40× water objective on the Opera Phenix Plus HCS System (Revvity). The high content cell analysis was performed using Harmony 5.1 software. To quantify DNA damage, nuclei were identified and quantified using Hoechst 33342 staining. The number of γH2AX foci was then determined within each identified nucleus. Afterwards, the total number of γH2AX foci was normalized by the number of nuclei in the well for variations in cell density. For synergy analysis, the data were normalized between 0% and 100%, with 0% being defined as the lowest response (vehicle), and 100%, defined as the highest response in that experimental replicate (i.e., highest observed DNA damage). The presented data denotes the mean of two independent experiments ± SEM performed in duplicate technical replicates.

### 4.6. CHD1L and PARP Trapping

CHD1L and PARP trapping were assessed as described previously in [18,32]. SW620 cells were plated at a density of 40,000 cells per well into clear bottom black 96-well plates (Revvity) coated with poly-L-Lysine and allowed to adhere overnight. Then, the cells were treated with different doses of OTI-611 or SN-38 and MMS to a final concentration of 0.001%. After 4 h of incubation, the cell media was aspirated and the cells were treated for 10 min at 4 °C with a cold cytoskeleton (CSK) buffer (10 mM PIPES pH = 6.8, 300 mM sucrose, 200 mM NaCl, 3 mM MgCl_2_) supplemented with 0.6% Triton X-100. The cells were then washed with cold PBS and fixed for 15 min at −20 °C with ice-cold methanol, followed by incubation with blocking solution (5% BSA in PBS) for 1 h at RT. A primary antibody (1:1000 CHD1L, 1:1000 PARP1 or 1:1000 PARP2) was then added and incubated O/N at 4 °C. Afterwards, the cells were incubated with a secondary antibody (goat anti-rabbit AlexaFluor 647, 1:500, or goat anti-mouse AlexaFluor 488, 1:500) for 1 h at RT. Finally, the cells were washed, stained with Hoechst 33342 (1:1000), and imaged using a 40× water objective on the Opera Phenix Plus HCS System (Revvity).

### 4.7. Immunofluorescence of Cell Death Markers

AIF translocation and caspase-3 activation were assessed by immunofluorescence. HCT116 cells were seeded into 96-well PhenoPlates (Revvity) and allowed to adhere overnight. The cells were then treated with different concentrations of OTI-611, SN-38, and their combination for 18 h. The media was then aspirated, and the cells were fixed with 4% paraformaldehyde for 15 min at RT. After washing twice with PBS, the cells were blocked in blocking buffer (5% BSA, 0.3% Triton-X in PBS) for 1 h at RT. Afterwards, the cells were washed twice with PBS and incubated with the corresponding primary antibody with the following indicated dilutions, timepoints, and temperatures: anti-AIF, 1:100, O/N, 4 °C; and cleaved caspase-3, 1:400, O/N, 4 °C. The cells were washed again and incubated with a secondary antibody for 1–2 h at RT (goat anti-rabbit AlexaFluor 647, 1:500). Finally, the cells were washed, stained with Hoechst 33342 (1:1000), and imaged using a 40× water objective on the Phenix Plus HCS System (Revvity). Nuclear AIF quantification was performed by identifying nuclei using Hoechst 33342 staining, and the mean intensity of AIF within the nuclear mask region was measured. To account for non-specific signal, the background intensity was calculated using a control well that was incubated without the primary antibody and subtracted from the measured AIF intensity. The background-subtracted nuclear AIF intensity was then divided by the number of nuclei in the field to obtain the final values. The quantification of the active caspase-3 was carried out by identifying and quantifying nuclei with Hoechst staining. The total active caspase-3 signal was determined by summing the intensities of all detected active caspase-3 signals in the field as determined by a cell mask. Afterwards, this value was normalized by the number of nuclei in the field of view to account for differences in cell numbers. Finally, the data presented in the graph was normalized to the control-treated cells (vehicle) for comparative analysis.

### 4.8. CRC Xenograft Studies

Athymic nude mice (6–7 weeks of age) were purchased from Envigo (Indianapolis, IN, USA) (Hsd Nude-Foxn1nu (069)). SW620 cells were cultured in RPMI 1640 (Gibco, Billings, MT, USA, cat no. 11875) supplemented with 5% FBS, then harvested and suspended at 2 × 10^6^ cells/100 µL in a 1:1 mixture of Matrigel (Corning, cat no. 356231) and RPMI 1640. Mice were anesthetized with 5% inhaled isoflurane and inoculated subcutaneously with 100 µL of the cell suspension into each flank. Two weeks later, the mice were randomized into four groups: vehicle (10% DMSO + 40% PEG400 in sterile PBS), OTI-611 (5 mg/kg), irinotecan (50 mg/kg), and OTI-611 (5 mg/kg) + irinotecan (50 mg/kg). All groups received IP administration, with the vehicle and OTI-611 groups treated daily. The mice receiving irinotecan (Selleckchem, #S2217) were given their doses once a week, starting four days after the initiation of the OTI-611 treatment. All groups were treated for 33 days. Body weights and tumor volumes were monitored twice weekly, with tumor volumes measured by caliper and calculated using the formula (L × W^2^)/0.5236. The mice were sacrificed when tumors reached a maximum volume of 2000 mm^3^ individually, or when the combined volume of the left and right tumors reached 3000 mm^3^. Two-way ANOVA was used to assess the significance between the groups.

### 4.9. Drug Synergy Evaluation

For the evaluation of the synergistic effect of OTI-611 in combination with chemotherapeutic agents in causing cytotoxicity or DNA damage, we used the Bliss synergistic score and Combination Sensitivity score calculated by the SynergyFinder R package version 3.12.0 [23,33]. The dose–response matrix graphs are interpreted as contour graphs, in which darker shades represent an increase in response (DNA damage) and lighter shades represent a decrease in response. The 3D contour Bliss Synergy score graphs are interpreted as darker shades of green representing an increase in synergy score. Drug synergy values less than −10 are considered antagonistic, values −10 to 10 are considered additive, and values above 10 are considered synergistic.

### 4.10. Statistical Analysis

The data were examined for statistical significance using GraphPad Prism 10, using one-way ANOVA tests, as indicated in the figure legends. All experiments were performed in 2–3 independent experimental replicates, and data are expressed as mean ± S.E.M. Significance levels were defined as follows: ns *p* > 0.05, * *p* < 0.05, ** *p* < 0.01, *** *p* < 0.001, **** *p* < 0.0001. IC_50_ values were calculated using GraphPad Prism 10, fitting the dose–response curves to a nonlinear regression model. Additional statistical analysis parameters can be found in the Supplementary Cytotoxicity File.

## 5. Conclusions

Our study demonstrates that the combination of CHD1Li OTI-611 with SOC chemotherapy significantly enhances treatment outcomes in CRC. We observed a striking synergistic antitumor effect in CRC cell lines and tumor organoids. Notably, OTI-611 effectively traps CHD1L on chromatin, leading to the induction of PARthanatos-programmed cell death. Additionally, in vivo studies using CRC xenograft mouse models indicate that the combination of OTI-611 and irinotecan not only reduces tumor progression but also extends survival by 30 days compared to irinotecan alone. This unprecedented synergy, characterized by substantial fold increases in chemotherapeutic potency, highlights OTI-611 as a promising candidate for future clinical development. Our findings provide a strong foundation for the further investigation of CHD1L inhibition in combination with chemotherapy and targeted therapies, with the potential to improve survival rates in CRC patients resistant to current treatments and to expand therapeutic strategies across a broader range of cancers.

## Data Availability

The data that support the findings of this study are included in this published article and its Supplementary Information files.

## Supplementary Materials

The following supporting information can be downloaded at: https://www.mdpi.com/article/10.3390/ijms252313160/s1.
