# Supplementary material for: CHD1L Inhibitor OTI-611 Synergizes with Chemotherapy to Enhance Antitumor Efficacy and Prolong Survival in Colorectal Cancer Mouse Models
Source: Int J Mol Sci. 2024 Dec 7;25(23):13160. doi: 10.3390/ijms252313160 (PMC11641930; doi:10.3390/ijms252313160)
Supplement: Supplementary file 1 [file ijms-25-13160-s001.zip › Sala et al Supplemental Information.pdf]

Supplemental Information

CHD1L inhibitor OTI-611 synergizes with chemotherapy to enhance antitumor efficacy and prolong survival in colorectal cancer mouse models

Rita Sala[a,1], Hector Esquer[a c,d,1], Timothy Kellett[a,1], Sophia Clune [a], Paul Awolade[a, c], Laura A. Pike[a], Qiong Zhou[a,c,d], Wells A. Messersmith[b,c,d], Daniel V. LaBarbera[a,c,d,]*

[a]Skaggs School of Pharmacy and Pharmaceutical Sciences, Department of Pharmaceutical Sciences

[b]School of Medicine, Division of Medical Oncology

[c]CU Anschutz Center for Drug Discovery

[d]University of Colorado Cancer Center

University of Colorado Anschutz Medical Campus, Aurora 80045, Colorado, U.S.A.

[1]R.S, H.E, T.K. contributed equally to this manuscript.

*Correspondence: daniel.labarbera@cuanschutz.edu

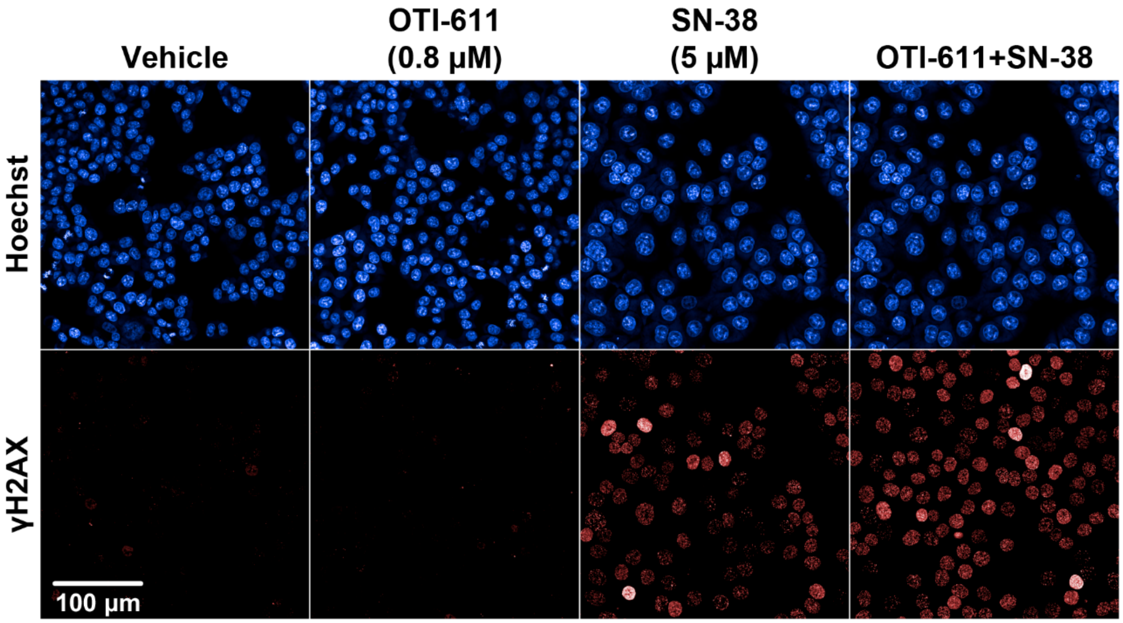

Supplemental Figure S 1. CHD1L inhibition potentiates DNA damage in HCT116 cells. Representative images of HCT116 cells treated with 5-FU in combination with OTI-611. Nuclei are stained with Hoechst 33342 (blue) and DNA damage is measured by γH2AX fluorescent signal (red). Scale 100 μm.

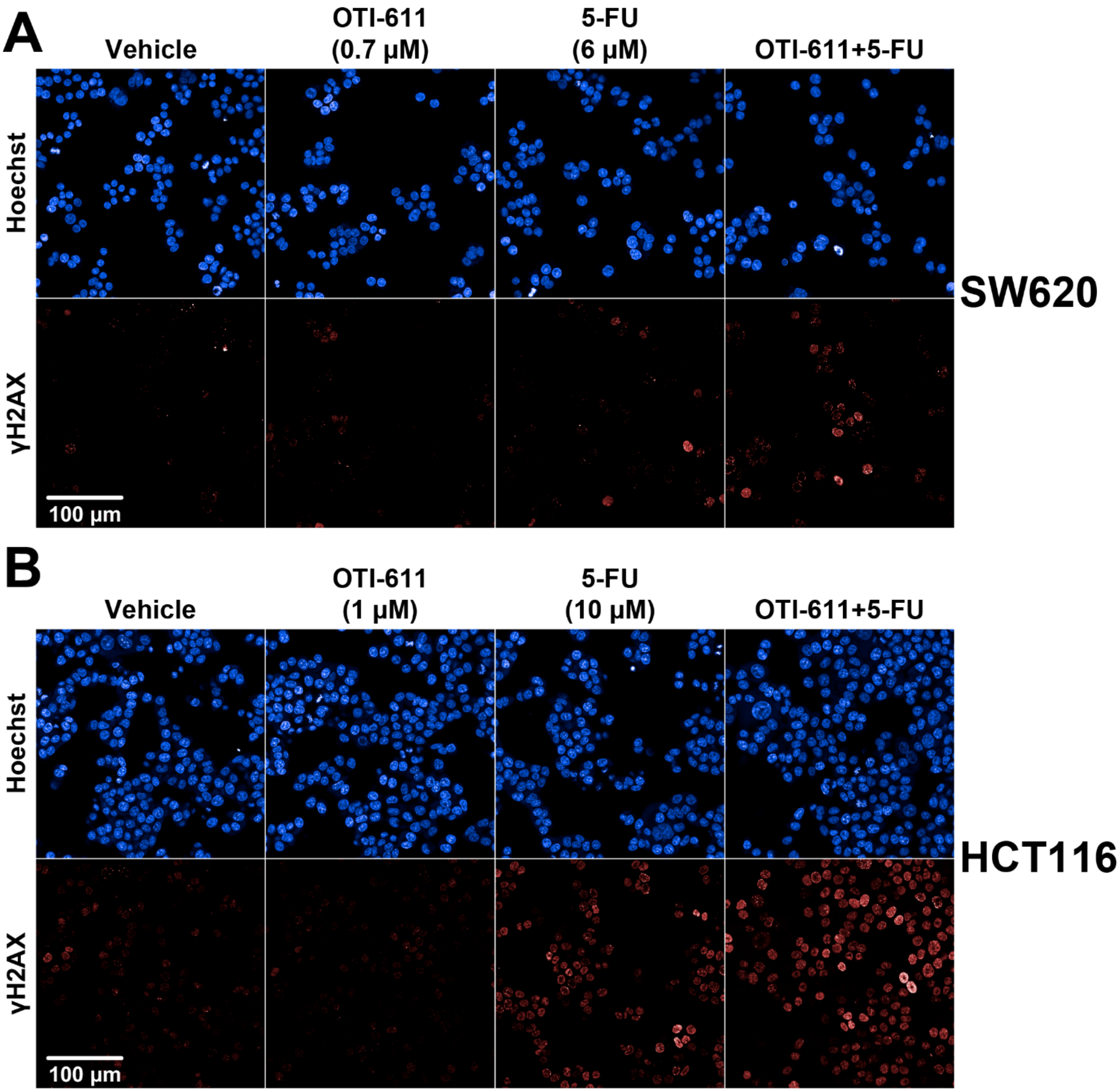

Supplemental Figure S 2. CHD1L inhibition potentiates DNA damage in CRC. Representative images of **(A)** SW620 and **(B)** HCT116 cells treated with 5-FU in combination with OTI-611. Nuclei are stained with Hoechst 33342 (blue) and DNA damage is measured by γH2AX fluorescent signal (red). Scale 100 µm.

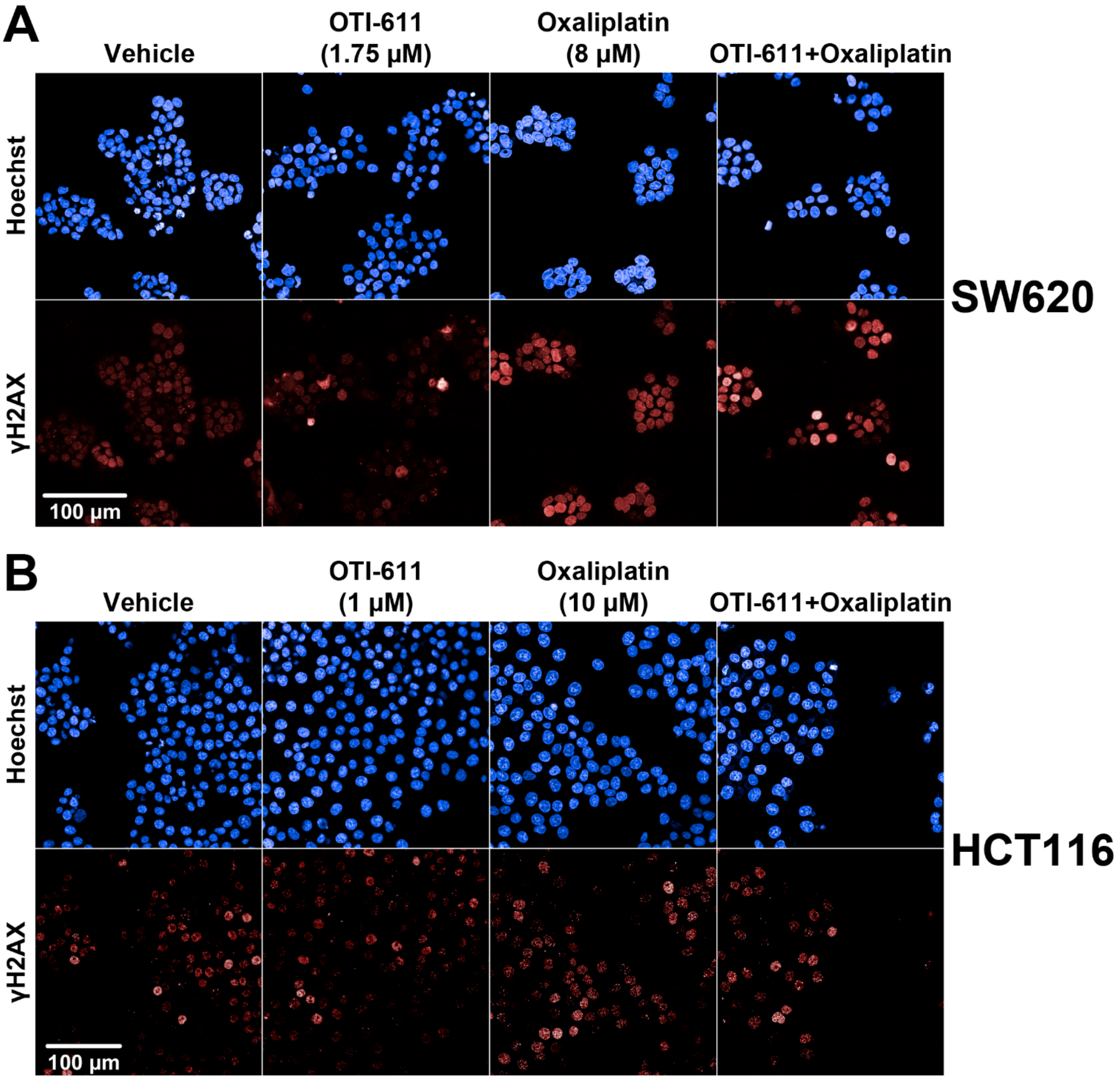

Supplemental Figure S3. CHD1L inhibition potentiates Oxaliplatin Induced DNA damage in SW620 and HCT116 cells. Representative images of **(A)** SW620 and **(B)** HCT116 cells treated with Oxaliplatin in combination with OTI-611. Nuclei are stained with Hoechst 33342 (blue) and DNA damage is measured by γH2AX fluorescent signal (red). Scale 100 µm.

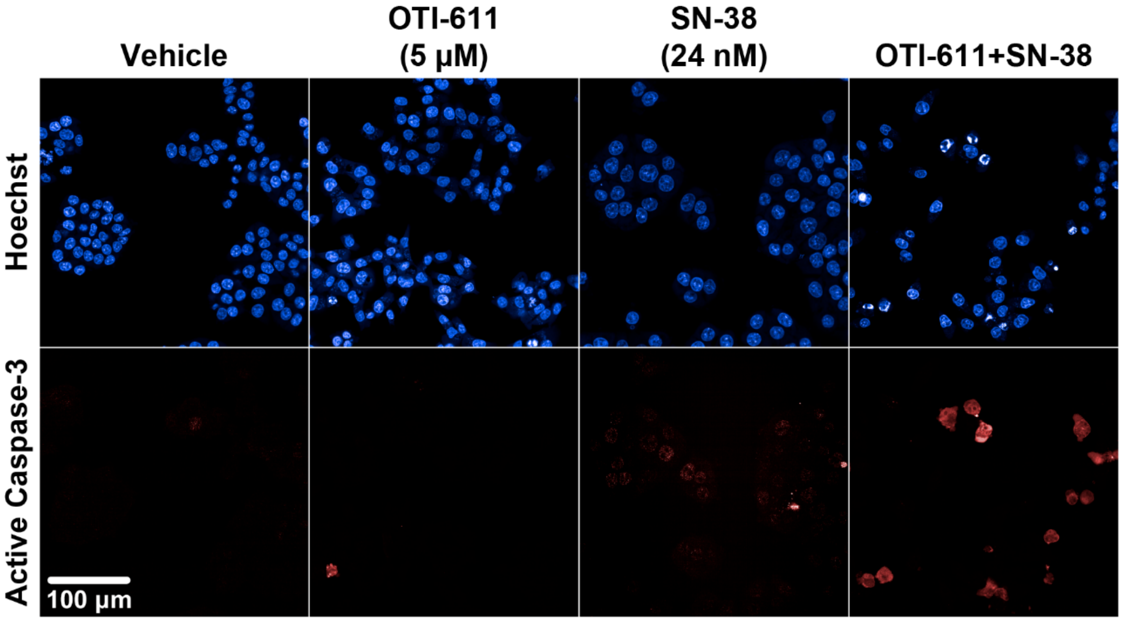

Supplemental Figure S4. OTI-611 does not induce an apoptotic cell death. Representative images of HCT116 cells treated with OTI-611, SN-38, and OTI-611 in combination with SN-38. Nuclei are stained with Hoechst 33342 (blue) and Caspase-3 fluorescent signal (red) was used as a marker of apoptotic cell death. Scale 100 μm.
